# Diminished Prolinemia in Chronic Chagasic Patients: A New Clue for Disease Pathology?

**DOI:** 10.3390/molecules24173167

**Published:** 2019-08-30

**Authors:** Sandra Carla Rocha, Ana Rosa Pérez, Juan Beloscar, Oscar Bottasso, Ariel Mariano Silber

**Affiliations:** 1 Laboratory of Biochemistry of Tryps—LaBTryps, Department of Parasitology, Institute of Biomedical Sciences, University of São Paulo, Av. Prof. Lineu Prestes 1374, Sao Paulo 05508000, Brazil; 2 Institute of Clinical and Experimental Immunology of Rosario (IDICER-CONICET UNR), Suipacha 590, Rosario 2000, Argentina

**Keywords:** prolinemia, proline, *Trypanosoma cruzi*, Chagas disease, cardiomyopathy, metabolism, pathology, collagen, fibrosis, severe

## Abstract

*Trypanosoma cruzi*, the etiological agent of Chagas disease, is dependent on proline for a variety of processes, such as energy metabolism, host cell invasion, differentiation, and resistance to osmotic, metabolic, and oxidative stress. On this basis, we investigated a possible relationship between prolinemia and severity of *T. cruzi* infection in chronic patients, as reported here. The study population consisted of 112 subjects, separated into 83 chronically *T. cruzi*-infected patients and 29 age-matched healthy volunteers (control) of both sexes, recruited at the Chagas Disease Service from the Department of Cardiology, Hospital Provincial del Centenario de Rosario (Rosario, Argentina). Chagasic patients were separated into three groups: chronic asymptomatic, mild/moderate, and severe chronic chagasic cardiomyopathy (CCC) subjects. We observed a significant decrease of 11.7% in prolinemia in chagasic patients when compared to controls. Further analysis within the three groups of chagasic patients also revealed a statistically significant decrease of prolinemia in severe CCC patients compared to controls, showing a relative difference of 13.6% in proline concentrations. These data point to the possibility that collagen—which participates in the healing process of cardiac tissue—and proline metabolism in the myocardium could constitute new factors affecting the evolution of Chagas disease.

## 1. Introduction

Chagas disease is a chronic and systemic infection caused by the hemoflagellate protozoan *Trypanosoma cruzi* that is usually transmitted to humans by triatomine insects. The disease is endemic in the Americas, with approximately 6–7 million people chronically infected and approximately 10,000 deaths per year [1,2]. Most chronically infected individuals present a chronic asymptomatic (CA) form of the disease without demonstrable pathology, which is characterized by the lack of clinical signs and symptoms of the chronic form of the disease. The CA form can evolve with a variable degree of organ involvement, mostly cardiomyopathy and gastrointestinal disorders. Chronic chagasic cardiomyopathy (CCC) is characterized by diffuse myocarditis with inflammatory infiltrate foci, cardiac fiber damage, and prominent fibrosis. This chronic phase of the disease is also characterized by a low but persistent parasite load, which may be responsible for chronic inflammatory reactivity at the myocardial level. Depending on its clinical severity, CCC can be classified as mild, moderate, or severe [2]. Moreover, factors accounting for such diverse disease outcomes involve a complex series of interactions between the host and parasite biology. In this regard, l-proline was shown to be essential in several aspects of *T. cruzi* biology, such as the development of its intracellular stage [3] and resistance to different stress conditions [4,5]. As reported in our previous studies, l-proline is also involved in energy supply and parasite survival in different environments [5,6]. Increased prolinemia (hyperprolinemia) was discovered as the cause of a metabolic syndrome in humans more than half a century ago, [7] and was classified as being type I or II depending on which enzyme is responsible for the deficiency (proline dehydrogenase causes type I hyperprolinemia, while P5C dehydrogenase causes type II hyperprolinemia) (reviewed by [8]). In addition, since hyperprolinemia started to be diagnosed, it was observed that values of prolinemia considered as physiological may vary in the range from 0.13 to 0.63 mM [9]. On this basis, we hypothesized a possible relationship between prolinemia and the severity of the *T. cruzi* infection. Accordingly, we have assessed the circulating levels of proline in sera from a series of chronic chagasic subjects lacking symptoms or coursing the infection with different degrees of severity of CCC.

## 2. Results

To compare proline contents among samples from chronic chagasic patients and uninfected individuals, participants were separated into four groups. Two of them consisted of *T. cruzi*-infected patients with mild/moderate severity (M/M, *n* = 26) and severe (Sev, *n* = 30) CCC, a third group was composed of asymptomatic infected subjects (CA, *n* = 27), and the fourth group consisted of healthy individuals (control group—Co, *n* = 29). Remarkably, among all the clinical parameters measured in those patients, glycemia was the only one that was increased when the Sev group was compared to the Co group (major group characteristics are shown in Table 1). 

**Figure 1:**
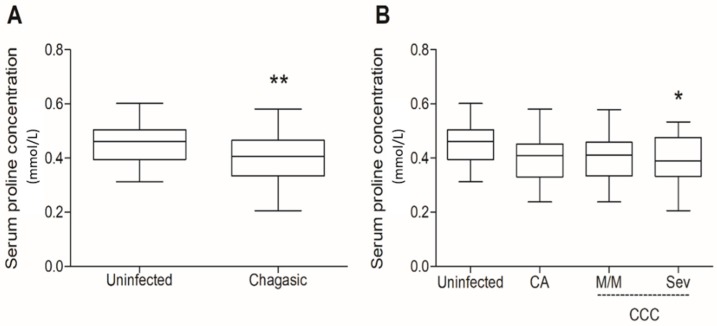
Serum proline in chagasic subjects. Serum samples were obtained from healthy and *T. cruzi*-infected patients from the Hospital Provincial del Centenario de Rosario (Rosario, Argentina). (**A**) All chagasic subjects (*n* = 83) were grouped and compared to uninfected healthy controls. (**B**) Chagasic subjects were divided into CA (*n* = 27), M/M (*n* = 26), and Sev (*n* = 30) groups and compared to controls. The control uninfected group was composed by *T. cruzi*-seronegative subjects (*n* = 29). The samples were submitted to a colorimetric test which uses the ninhydrin as proline marker (see Materials and Methods). The reading was carried out at 515 nm in a spectrophotometer. CCC, chronic chagasic cardiomyopathy; CA, chronic asymptomatic; M/M, mild and moderate; Sev, severe. Box plots show median values, 25–75th percentiles, and maximum and minimum values of the data from each group. The statistics were obtained by T-test (**A**), one-way ANOVA, and Tukey’s multiple comparison test (**B**), with differences being regarded as statistically different when *p* < 0.05. * *p* < 0.05; ** *p* < 0.01

Subjects from the CA group had normal electrocardiograms (ECGs), chest X-rays, and routine laboratory test results. The M/M group was composed of individuals with mild or moderate cardiac compromise and no congestive heart failure, but pathological ECG tracings such as complete or incomplete right bundle branch block or ventricular arrhythmia and a chest X-ray cardiothoracic ratio of <0.55. The Sev group consisted of patients presenting congestive heart failure, pathological ECG tracings, and a chest X-ray cardiothoracic ratio of >0.55 with normal CPK levels. Noteworthily, glycemia was slightly enhanced in these patients. Samples from the control group had a mean prolinemia value of 0.454 ± 0.014 mmol/L (means ± SE, *n* = 29), whereas the overall group of chagasic patients showed values of 0.401 ± 0.01 mmol/L (means ± SE, *n* = 83). Therefore, prolinemia was reduced by 11.7% in infected patients, which was statistically significant when compared to controls (*p* = 0.0054) (for individual values of prolinemia, see Table S1). Considering each group individually, differences reached the level of statistical significance when the prolinemia values from the Co group were compared with those of Sev patients, which presented a 13.6% decrease in proline concentrations (0.392 ± 0.018 mmol/L, *p* < 0.05, Figure 1). Further analysis within the groups of chagasic patients revealed no significant differences among them (for descriptive statistics of the prolinemia data, see Table S2).

## 3. Discussion

The variability and severity of the symptoms of Chagas disease depend on many factors, such as the parasite strain [10], parasite load, infection route [2], host immunity [11], and number of reinfections [12]. In addition, the parasite persistence at specific sites of the infected host induces a local immune response, which is responsible for tissue damage, especially of myocardial cells [11]. CCC is characterized by inflammation, fibrosis, myocytolysis, vasculitis, and parasitic persistence, leading to different degrees of clinical manifestations, i.e., mild, moderate, or severe [2]. Our findings point to a possible association between prolinemia and disease pathology during *T. cruzi* infection. Considering that the results were different from our initial hypothesis, we reinforce the suggestion that the diminished prolinemia in Sev subjects can be related to the clinical evolution of Chagas disease. As previously mentioned, the reported serum proline concentration in healthy individuals is within the range of 0.13 and 0.63 mmol/L [9]. We also observed a slight increase in glycemia in the Sev group with respect to uninfected controls. It has been previously shown that proline can be a precursor for gluconeogenesis [13] and that lactate is an allosteric regulator of proline oxidase (the first enzyme in the proline oxidation pathway). On this basis, it was suggested that lactate may influence the hepatic availability of proline, which can be used for glucose biosynthesis, resulting in increased glycemia [14]. Further investigations are necessary to confirm the existence of a link between these two metabolic parameters. The relationship between prolinemia and some types of disease also has been studied in cancer research. Decrease of serum proline has been related to renal cell carcinoma [15], oral cancer [16], colorectal cancer [17], and esophageal cancer [9]. Some of these studies suggest that the lower level of serum proline could be an indication of overutilization of amino acids in tumor tissue [9]. 

Prolidase (E.C.3.4.13.9) is a cytosolic exopeptidase that is widely distributed in humans and animals, which splits imidodipeptides (originated from the degradation of procollagen, collagen, and proteins) into free proline or hydroxyproline within the cytoplasm [18]. In addition to its primary biological function in the collagen degradation metabolism, prolidase also participates in the recycling of proline from dipeptides to resynthetize collagen [19]. Thus, plasma prolidase activity might be a possible indicator of collagen catabolism, as already shown in chronic liver disease [18]. Furthermore, Myara et al. [19] suggest that plasma prolidase activity might be high in the early stage of fibrosis and might subsequently drop in an advanced fibrosis stage. This way, the disturbance of the collagen metabolism could interfere with the free proline concentration. This idea is consistent with previous findings showing that fibroblasts obtained from prolidase-deficient subjects had higher collagen degradation rates and lower proline levels when compared to control cells [20]. Moreover, it was recently suggested that a decrease in prolidase activity is an indicator of advanced fibrosis [21]. 

Regarding Chagas disease, it is known that severe CCC patients usually have myocardial fibrosis, which is defined also by the progressive accumulation of fibrillar extracellular matrix (ECM) in this tissue. These changes occur as a repair mechanism in response to chronic cardiac damage caused by several factors, including an intense inflammatory response (reviewed by [2]). Considering this background, we propose the following: (i) Severe CCC patients have diminished prolinemia because proline is the main substrate for the synthesis of collagen destined for the reparative process of cardiac tissue. Thus, the extension of myocardial fibrosis would be inversely related to the serum proline amount. (ii) Plasma prolidase activity might decrease in advanced fibrosis compared to the early stage of fibrosis, leading to a decrease in free proline in the tissue fluids. This phenomenon probably stems from decreased collagen turnover, which in turn is secondary to decreased functional heart tissue [22]. In summary, our results point to collagen and proline metabolism in the myocardium as being new factors to be investigated to gain better insight into the physiopathology of Chagas disease. 

## 4. Material and Methods

### 4.1. Study Design and Participants

The study population consisted of 112 subjects, separated into 83 chronically *T. cruzi*-infected patients and 29 age-matched healthy volunteers (control) of both sexes, recruited at the Chagas Disease Service from the Department of Cardiology, Hospital Provincial del Centenario de Rosario (Rosario, Argentina). None of these patients were under specific treatment (i.e., benznidazole or nifurtimox), and none had concomitant pathological disorders. Exclusion criteria comprised neuroendocrine disturbances, metabolic diseases (such as diabetes), immunological diseases, and treatment with hormones or immunomodulators. Control subjects were seronegative to *T. cruzi*-specific tests. The diagnosis was based on at least two positive serological findings (either by ELISA, hemagglutination, or immunofluorescence), together with clinical symptoms, chest X-rays, and 12-lead resting electrocardiograms (ECGs). Routine laboratory, glycemia, and creatine phosphokinase (CPK) parameters were also assayed.

### 4.2. Determination of Serum Proline Levels in Chagasic Subjects

Serum proline quantification was performed as described previously [23]. Briefly, samples were diluted 1:5 and 1:10 in distilled water. Volumes of 250 μL of the diluted samples were incubated with 250 μL of glacial acetic acid and 250 μL of ninhydrin solution (0.25 g ninhydrin diluted in a mixture of 6 mL of glacial acetic acid and 4 mL of 6 M phosphoric acid) for 1 h at 100 °C. Then, the samples were incubated on ice for at least 1 min. The organic solutes (including those that reacted with ninhydrin) were extracted by partitioning with 500 μL of toluene. The organic phase in each sample was separated by centrifugation (1 min, 10,000× *g*), and 100 μL of the organic phase was transferred to a 96-well polypropylene plate. Prolinemia in each sample was determined in four replicas. The absorbance was measured in a spectrophotometer at 515 nm and compared with a calibration curve in which known proline concentrations were used as standards. The prolinemia values were expressed as mmol/L.

### 4.3. Statistical Analyses

The data were processed using GraphPad Prism software version 4.0. The normality of population distribution was evaluated by using a nonparametric Kolmogorov–Smirnov test. The significant differences were evaluated by using one-way ANOVA, followed by the Tukey post-test for the multiple comparison approach or the chi-squared test (χ^2^). The Student’s t-test was used when only two groups were compared. Statistical differences with *p* < 0.05 were considered significant.

### 4.4. Ethics Statement

The study was conducted according to the Declaration of Helsinki, and the protocol was approved by the Institutional Ethics Committee of the Medical Faculty of National University of Rosario. Participants were enrolled and gave their informed consent before inclusion. All human subjects were adults. The protocol was approved under the resolution number 4977/2013.

## Figures and Tables

**Table 1:** Characteristics of the groups of patients included in the study.

			CCC		
	Co	CA	M/M	Sev	*p* vs. Co
Age (Range) in Years	43 (18–70)	45 (18–76)	50 (21–76)	53 (19–79)	
Sex, Males/Females	10/16	13/14	10/16	13/17	
*Biochemical determinations*					
Leukocytes/(mm^3^) RV: 4–9 × 10^3^	6.5 ± 0.5 × 10^3^	7.0 ± 0.2 × 10^3^	7.5 ± 0.3 × 10^3^	6.9 ± 0.4 × 10^3^	
Lymphocytes/(%) RV: 20–50	35 ± 2	33 ± 2	34 ± 3	31 ± 1	
Total protein (g/dL) RV: 6.6–8.7	7.5 ± 0.1	8.0 ± 0.2	7.7 ± 0.2	7.4 ± 0.3	
Albumin/(g/dL) RV: 3.4–4.8	4.4 ± 0.1	4.4 ± 0.0	4.3 ± 0.0	4.3 ± 0.1	
Glycemia (mg/dL) RV: 70–100	82 ± 10	87 ± 13	89 ± 12	93 ± 13 *	<0.05
*Cardiac parameters*					
CPK RV: 0–170	105 ± 8	110 ± 5	123 ± 8	128 ± 5	
Systolic blood pressure (mm Hg)	120 ± 1.2	120 ± 1.8	128 ± 2.2	131 ± 1.4	
Diastolic blood pressure (mm Hg, mean/SEM)	7.0 ± 1.6	7.4 ± 1.2	7.7 ± 1.3	8.1 ± 1.3	

Data were expressed as the mean ± SEM (SEM: Standard Error of the Mean), except for age (median/rank). * *p* < 0.05.

## Supplementary Materials

The following are available online at https://www.mdpi.com/1420-3049/24/17/3167#supplementary, Table S1: Individual prolinemia data; Table S2: Descriptive statistics of prolinemia data.

## References

[1] World Health Organization Chagas Disease (American Trypanosomiasis) Home Page. http://www.who.int/mediacentre/factsheets/fs340/en/.

[2] Rassi A., Rassi A., Marin-Neto J.A. (2010). Chagas disease. Lancet.

[3] Silber A.M., Tonelli R.R., Lopes C.G., Cunha-e-Silva N., Torrecilhas A.C.T., Schumacher R.I., Colli W., Alves M.J.M. (2009). Glucose uptake in the mammalian stages of *Trypanosoma cruzi*. Mol. Biochem. Parasitol..

[4] Magdaleno A., Ahn I.Y., Paes L.S., Silber A.M. (2009). Actions of a proline analogue, l-thiazolidine-4-carboxylic acid (T4C), on *Trypanosoma cruzi*. PLoS ONE.

[5] Paes L.S., Suárez Mantilla B., Zimbres F.M., Pral E.M.F., Diogo de Melo P., Tahara E.B., Kowaltowski A.J., Elias M.C., Silber A.M. (2013). Proline Dehydrogenase Regulates Redox State and Respiratory Metabolism in *Trypanosoma cruzi*. PLoS ONE.

[6] Mantilla B.S., Paes L.S., Pral E.M.F., Martil D.E., Thiemann O.H., Fernández-Silva P., Bastos E.L., Silber A.M. (2015). Role of Δ1-pyrroline-5-carboxylate dehydrogenase supports mitochondrial metabolism and host-cell invasion of *Trypanosoma cruzi*. J. Biol. Chem..

[7] Mitsubuchi H., Nakamura K., Matsumoto S., Endo F. (2008). Inborn Errors of Proline Metabolism. J. Nutr..

[8] Schafer I.A., Scriever C.R., Efron M.L. (1962). Familial hyperprolinemia, cerebral dysfunction and renal anomalies occurring in a family with hereditary nephropathy and deafness. New Engl. J. Med..

[9] Liang S., Sanchez-Espiridion B., Xie H., Ma J., Wu X., Liang D. (2015). Determination of proline in human serum by a robust LC-MS/MS method: Application to identification of human metabolites as candidate biomarkers for esophageal cancer early detection and risk stratification. Biomed. Chromatogr..

[10] Vago A.R., Andrade L.O., Leite A.A., d’Ávila Reis D., Macedo A.M., Adad S.J., Tostes S., Moreira M.C., Filho G.B., Pena S.D.J. (2000). Genetic characterization of *Trypanosoma cruzi* directly from tissues of patients with chronic chagas disease: Differential distribution of genetic types into diverse organs. Am. J. Pathol..

[11] Teixeira A.R.L., Hecht M.M., Guimaro M.C., Sousa A.O., Nitz N. (2011). Pathogenesis of chagas’ disease: Parasite persistence and autoimmunity. Clin. Microbiol. Rev..

[12] Bustamante J.M., Rivarola H.W., Fernández A.R., Enders J.E., Fretes R., Palma J.A., Paglini-Oliva P.A. (2002). *Trypanosoma cruzi* reinfections in mice determine the severity of cardiac damage. Int. J. Parasitol..

[13] Greth W.E., Their S.O., Segal S. (1978). The transport and metabolism of l-proline-14C in the rat in vivo. Metabolism.

[14] Kowaloff E.M., Phang J.M., Granger A.S., Downing S.J. (1977). Regulation of proline oxidase activity by lactate. Proc. Natl. Acad. Sci. USA.

[15] Mustafa A., Gupta S., Hudes G.R., Egleston B.L., Uzzo R.G., Kruger W.D. (2011). Serum amino acid levels as a biomarker for renal cell carcinoma. J. Urol..

[16] Tiziani S., Lodi A., Khanim F.L., Viant M.R., Bunce C.M., Günther U.L. (2009). Metabolimic profiling of drug responses in acute myeloid leukemia cell lines. PLoS ONE.

[17] Qiu Y., Cai G., Su M., Chen T., Zheng X., Xu Y., Ni Y., Zhao A., Xu L.X., Cai S. (2009). Serum metabolite profiling of human colorectal cancer using GC-TOFMS and UPLC-QTOFMS. J. Proteome Res..

[18] Myara I., Myara A., Mangeot M., Fabre M., Charpentier C., Lemonnier A. (1984). Plasma prolidase activity: A possible index of collagen catabolism in chronic liver disease. Clin. Chem..

[19] Jackson S.H., Heininger J.A. (1975). Proline recycling during collagen metabolism as determined by concurrent 18O2- and 3H-labeling. BBA-Gen. Subj..

[20] Chamson A., Voigtlander V., Myara I., Frey J. (1989). Collagen biosynthesis anomalies in prolidase deficiency: Effect of glycyl-l-proline on the degradation of newly synthesized collagen. Clin. Physiol. Biochem..

[21] Kitchener R.L., Grunden A.M. (2012). Prolidase function in proline metabolism and its medical and biotechnological applications. J. Appl. Microbiol..

[22] Sezen Y., Bas M., Altiparmak H., Yildiz A., Buyukhatipoglu H., Dag O.F., Kaya Z., Aksoy N. (2010). Serum prolidase activity in idiopathic and ischemic cardiomyopathy patients. J. Clin. Lab. Anal..

[23] Bates L.S., Waldren R.P., Teare I.D. (1973). Rapid determination of free proline for water-stress studies. Plant Soil.

