# Supplementary material for: Diminished Prolinemia in Chronic Chagasic Patients: A New Clue for Disease Pathology?
Source: Molecules. 2019 Aug 30;24(17):3167. doi: 10.3390/molecules24173167 (PMC6749509; doi:10.3390/molecules24173167)
Supplement: Supplementary file 1 [file molecules-24-03167-s001.pdf]

Table S1: Individual prolinemia data

		CCC	
UNIN	IND	M/M	SEV
0.487	0.424	0.484	0.482
0.460	0.581	0.422	0.499
0.446	0.537	0.483	0.478
0.378	0.359	0.424	0.412
0.601	0.452	0.319	0.462
0.402	0.411	0.440	0.531
0.419	0.316	0.548	0.387
0.435	0.350	0.510	0.382
0.385	0.410	0.450	0.264
0.425	0.327	0.339	0.433
0.466	0.314	0.349	0.463
0.396	0.382	0.278	0.389
0.339	0.424	0.533	0.364
0.493	0.438	0.579	0.331
0.559	0.500	0.442	0.466
0.487	0.331	0.433	0.406
0.517	0.560	0.310	0.475
0.378	0.437	0.296	0.534
0.421	0.406	0.399	0.495
0.312	0.293	0.399	0.373
0.332	0.439	0.269	0.382
0.596	0.356	0.405	0.206
0.473	0.514	0.399	0.252
0.484	0.238	0.238	0.351
0.487	0.473	0.417	0.527
0.516	0.397	0.346	0.363
0.549	0.319		0.334
0.517			0.219
0.394			0.277
			0.214

Prolinemia data are individually presented as millimol/Liter.

Abbreviations: CCC, chronic chagas cardiomyopathy; IND, indeterminate; M/M, mild and moderate; SEV, severe; UNIN, uninfected

Table S2: Descriptive statistics of prolinemia data

Number of values	29	27	26	30
Minimum	0.312	0.238	0.238	0.206
25% Percentile	0.395	0.331	0.334	0.3333
Median	0.46	0.41	0.411	0.388
75% Percentile	0.5045	0.452	0.4583	0.4758
Maximum	0.601	0.581	0.579	0.534
Mean	0.4536	0.407	0.4043	0.3917
Std. Deviation	0.07504	0.085	0.08912	0.0976
Std. Error	0.01393	0.01636	0.01748	0.01782
Lower 95% CI of mean	0.425	0.3733	0.3683	0.3553
Upper 95% CI of mean	0.4821	0.4406	0.4403	0.4281
Sum	13.15	10.99	10.51	11.75

Data are presented as millimol/Liter.
